# Identification of the angiogenic gene signature induced by EGF and hypoxia in colorectal cancer

**DOI:** 10.1186/1471-2407-13-518

**Published:** 2013-11-02

**Authors:** Tak L Khong, Ngayu Thairu, Helene Larsen, Peter M Dawson, Serafim Kiriakidis, Ewa M Paleolog

**Affiliations:** 1 Kennedy Institute of Rheumatology, Faculty of Medicine, Imperial College, London, UK; 2 Department of Surgery and Cancer, Faculty of Medicine, Imperial College, London, UK; 3 Present address: Kennedy Institute of Rheumatology, Nuffield Department of Orthopaedics, Rheumatology and Musculoskeletal Sciences, University of Oxford, 65 Aspenlea Road, London W6 8LH, UK

**Keywords:** Colorectal cancer, Angiogenesis, Hypoxia, EGF

## Abstract

**Background:**

Colorectal cancer (CRC) is characterised by hypoxia, which activates gene transcription through hypoxia-inducible factors (HIF), as well as by expression of epidermal growth factor (EGF) and EGF receptors, targeting of which has been demonstrated to provide therapeutic benefit in CRC. Although EGF has been demonstrated to induce expression of angiogenic mediators, potential interactions in CRC between EGF-mediated signalling and the hypoxia/HIF pathway remain uncharacterised.

**Methods:**

PCR-based profiling was applied to identify angiogenic genes in Caco-2 CRC cells regulated by hypoxia, the hypoxia mimetic dimethyloxallylglycine (DMOG) and/or EGF. Western blotting was used to determine the role of HIF-1alpha, HIF-2alpha and MAPK cell signalling in mediating the angiogenic responses.

**Results:**

We identified a total of 9 angiogenic genes, including angiopoietin-like (ANGPTL) 4, ephrin (EFNA) 3, transforming growth factor (TGF) β1 and vascular endothelial growth factor (VEGF), to be upregulated in a HIF dependent manner in Caco-2 CRC cells in response to both hypoxia and the hypoxia mimetic dimethyloxallylglycine (DMOG). Stimulation with EGF resulted in EGFR tyrosine autophosphorylation, activation of p42/p44 MAP kinases and stabilisation of HIF-1α and HIF-2α proteins. However, expression of 84 angiogenic genes remained unchanged in response to EGF alone. Crucially, addition of DMOG in combination with EGF significantly increased expression of a further 11 genes (in addition to the 9 genes upregulated in response to either DMOG alone or hypoxia alone). These additional genes included chemokines (CCL-11/eotaxin-1 and interleukin-8), collagen type IV α3 chain, integrin β3 chain, TGFα and VEGF receptor KDR.

**Conclusion:**

These findings suggest that although EGFR phosphorylation activates the MAP kinase signalling and promotes HIF stabilisation in CRC, this alone is not sufficient to induce angiogenic gene expression. In contrast, HIF activation downstream of hypoxia/DMOG drives expression of genes such as ANGPTL4, EFNA3, TGFβ1 and VEGF. Finally, HIF activation synergises with EGF-mediated signalling to additionally induce a unique sub-group of candidate angiogenic genes. Our data highlight the complex interrelationship between tumour hypoxia, EGF and angiogenesis in the pathogenesis of CRC.

## Background

Colorectal cancer (CRC) is the third most common cancer worldwide, with an estimated 530,000 patients dying from the condition each year [1]. Biological changes underlying malignant transformation are complex, but key events such as angiogenesis, induced in part by alterations in oxygen tension and growth factors, represent critical milestones in tumour progression, self-preservation and survival [2,3]. Low oxygen tension (hypoxia) plays a pivotal role in cancer, and low intra-tumoural oxygen tensions (below 30 mmHg, approximately 4% O_2_) have been demonstrated in many solid tumours, including CRC [4,5]. The Hypoxia Inducible Factor (HIF) family of transcription factors is central to the homeostatic mechanisms involved in the cellular response to hypoxic stress, regulating genes involved in nutritional stress, tumour metabolism, invasion, cell death and angiogenesis, including the key angiogenic molecule vascular endothelial growth factor (VEGF) [6,7]. Levels of HIF proteins increase in hypoxic conditions (generally at below 5% O_2_) due to increased stability, as a consequence of the inactivity of oxygen-dependent HIF hydroxylase enzymes [8-10]. In CRC, increased HIF expression correlates with carcinogenesis [11,12], tumour and lymphovascular invasion, liver metastasis [13] and VEGF expression [14], as well as with more advanced tumour stage at diagnosis and poorer prognosis [15]. Furthermore, Imamura *et al.* reported a statistically significant correlation between HIF-1α expression and both VEGF and microvessel density [16], while both Yoshimura *et al.* and Cleven *et al.* found poor prognosis to correlate with increased HIF-2α [17,18].

In addition to the important role of hypoxia/HIF in CRC, over-expression of epidermal growth factor (EGF) receptor (EGFR/HER-1) has been demonstrated in approximately 70-75% of CRC [19]. EGF signalling is not only capable of potent mitogenic and tumourigenic effects, but also stimulates angiogenesis in human solid tumours [20], through direct effects upon the endothelium of new vessels [21], or indirectly by altering expression of positive and negative regulators of angiogenesis by tumours. For example, studies with glioma, gastric and prostate cancer cells demonstrated increased VEGF expression following EGFR stimulation [20,22,23]. Conversely, inhibition of EGFR with antibodies or tyrosine kinase inhibitors resulted in abrogation of neovascularisation by downregulating VEGF and interleukin-8 (IL8) through repression of phosphoinositide 3-kinase (PI3K)/Akt signalling [23-25]. Furthermore, animal models have confirmed the inhibitory effects of EGFR antagonists, and these favourable results have been translated to the clinical application in metastatic CRC of therapies targeting EGFR, namely the monoclonal antibodies cetuximab [26,27] and panitumumab [28]. Crucially, HIFs are also regulated by growth factor signalling, for example EGF, suggesting that signalling cascades which play key roles in CRC – namely EGFR activation and HIFs – may converge. Increased HIF-1α protein and transcriptional activity following EGFR stimulation in various cell lines [29,30] was shown to be dependent upon activation of receptor tyrosine kinases and downstream PI3K/Akt/MTOR [31-33]. However, the regulation of HIFs by EGFR signalling in CRC, and the relative importance of the contributions of HIFs towards a global angiogenic response following EGFR activation, remain unexplored. Furthermore, given that EGFR over-activity and hypoxia are common features of solid tumours [19,34], it is conceivable that they may interact to modulate expression of HIFs and thus affect angiogenic gene responses in CRC.

In this study, we investigated whether EGF activated HIF signalling in Caco-2 CRC cells. Caco-2 CRC cells are an adherent cell line isolated from a patient with colorectal adenocarcinoma. These cells express functional wild-type EGFR [35], demonstrate responses to hypoxia through HIF-1 and HIF-2 regulation [10], and are frequently used as an *in vitro* model of CRC [36]. Furthermore, we examined the expression of a panel of angiogenic genes following EGFR activation, to elucidate the importance of HIF recruitment in mediating angiogenic responses following EGFR activation. We found that the HIF pathway was activated in Caco-2 CRC cells following exposure to EGF, and in response to hypoxia and the hypoxia mimetic dimethyloxalylglycine (DMOG). PCR array profiling generated a distinctive angiogenic gene signature in response to hypoxia alone or DMOG alone, with induction of angiopoietin (ANGPT) 1, angiopoietin like (ANGPTL) 3, ANGPTL4, ephrin (EFN) A1, EFNA3, FLT1, matrixmetalloprotease (MMP) 9, transforming growth factor (TGF) β1 and VEGF. No difference was observed between gene profiles induced by hypoxia *versus* the hypoxia mimetic DMOG. We further characterised the 4 candidate genes which were upregulated to the greatest extent by hypoxia/DMOG – namely ANGPTL4, EFNA3, TGF β1 and VEGF - to be hypoxia-regulated in Caco-2 through the HIF-1α isoform. However, despite our observation that EGF activated receptor autophosphorylation, HIF stabilisation and p42/p44 MAPK signalling, angiogenic genes were unaltered by addition of EGF alone. In contrast, addition of a combination of DMOG and EGF did not further affect expression of the hypoxia/DMOG-regulated angiogenic gene signature, but these combined stimuli significantly upregulated expression of 11 additional angiogenic genes. These findings suggest that although EGF promotes HIF stabilisation in CRC, this is not sufficient to induce angiogenic gene responses. In contrast, hypoxia and EGF synergise to additionally induce a unique sub-group of candidate angiogenic genes, highlighting the complexity of the angiogenic process in CRC.

## Methods

### Experimental protocols

Caco-2, a moderately differentiated adherent CRC cell line (ATCC; Rockville, MD, USA) known to have non-transformed EGFR [35] and HIF pathways [10], were cultured in Eagle’s Minimum Essential Medium (EMEM) (Biowhittaker, Lonza, Switzerland) containing non-essential amino acids and 1 mM sodium pyruvate. Medium was supplemented with 1 mM Glutamine, 10% foetal bovine serum (FBS), 100 U/mL streptomycin and 1.1 μg/mL penicillin. For the experiments, Caco-2 cells were plated in the above medium until cells achieved 50% confluence. Cells were cultured for 24 hours in hypoxia (1% oxygen) using a Galaxy R Incubator (Wolf Laboratories, York, UK) or exposed to DMOG (dimethyloxaloylglycine; Biomol, Plymouth Meeting, PA, USA), a cell-permeable PHD inhibitor. Recombinant human EGF was purchased from Peprotech, Rocky Hill, NJ, USA.

For transfection studies, Caco-2 cells (50% confluence) were exposed to Lipofectamine and siRNA diluted in Opti-MEM (Invitrogen, Carlsbad, CA, USA) for 6 hours in serum-free EMEM. Subsequently, cells were supplemented with FBS, Glutamine and streptomycin/penicillin. After a further 18 hours, cells were exposed to either 1% O_2_ or 1 mM DMOG for 24 hours. siRNA sequences were purchased from MWG (Ebersberg, Germany) and siLuc was used as an irrelevant control: siHIF-1α 5′-[agcaguaggaauuggaacauu]RNA [tt]DNA 3′, siHIF-2α 5′-[gcgacagcuggaguaugaauu]RNA [tt]DNA 3′, siLuc 5′-[cguacgcggaauacuucga]RNA [tt]DNA 3′.

### Analysis of gene expression by quantitative polymerase chain reaction (Q-PCR)

RNA was extracted using the QIAamp RNA blood mini kit (QIAGEN, GmbH, Germany) according to the manufacturer’s protocol, followed by Turbo DNAse treatment (Ambion, Austin, USA). cDNA was synthesised using MMLV reverse transcriptase, RNase H Minus, Point Mutant (M-MLV RT (H-) and OligoDT primers (Promega, Madison, USA). Subsequently, PCR was performed using deoxynucleotide triphosphates (dNTPs), forward and reverse primers and SYBR® Green JumpStart™ *Taq* ReadyMix™ (Sigma-Aldrich, St Louis, MO, USA). The primers were manufactured by MWG Biotech (Ebersberg, Germany): acidic ribosomal phosphoprotein (ARP) Fwd: 5′-cgacctggaagtccaactac-3′, Rev: 5′-atctgctgcatctgcttg-3′; HIF-1α: Fwd: 5′-cacctctggacttgcctttc-3′, Rev: 5′-ggctgcatctcgagactttt-3; HIF-2α: Fwd: 5′-ccttcaagacaaggtctgca-3′, Rev: 5′-ttcatccgtttccacatcaa-3′; VEGF: Fwd: 5′-cttgccttgctgctctacct-3′, Rev: 5′-ctgcatggtgatgttggact-3′; ANGPTL4: Fwd: 5′-ccacttgggaccaggatcac-3′, Rev: 5′-cggaagtactggccgttgag-3′; EFNA3: Fwd: 5′-cactctcccccagttcaccat-3, Rev: 5′-cgctgatgctcttctcaagct-3′; TGFβ1: Fwd: 5′-gcaacaattcctggcgatac-3, Rev: 5′-aagccctcaatttcccctc-3′; 18S Fwd: 5′-gtaacccgttgaacccca-3′, Rev: 5′-ccatccaatcggtagtagcg-3′.

The amplification, detection and quantification steps were carried out using the Rotor-Gene 6000 centrifugal thermal cycler (Corbett Research Mortlake, Sydney, Australia). Gene expression was quantified using cycle threshold (C_t_) values by the comparative 2^-∆∆Ct^ method [37], normalised to the housekeeping gene (HKG) 18S. Comparable data were obtained when ARP was used as HKG (not shown).

### Analysis of gene expression by PCR-based angiogenesis arrays

The Human Angiogenesis RT^2^ Profiler™ PCR Array (SABiosciences, Frederick, MD, USA) was used to profile the expression of 84 key genes involved in angiogenesis, with cDNA synthesised using the RT^2^ First Strand Kit (SABiosciences, Frederick, MD, USA) according to the manufacturer’s instructions. RNA from 3 experiments was reverse transcribed and equal quantities of the generated cDNA were pooled. Each experimental condition was tested on duplicate PCR arrays using the ABI PRISM 7700 Sequence Detector (Foster City, CA, USA). Relative expression of various genes was calculated by the 2^-∆∆Ct^ comparative method. Data were normalised against the following HKG: 18S ribosomal RNA, 60S ribosomal protein L13a (RPLP13A), β-actin (ActB) and hypoxanthine phosphoribosyltransferase 1 (HPRT1). A gene expression fold-change threshold of 2.0 was applied, as previously described by our laboratory [38]. Arrays were performed in duplicate on 2 separate occasions using pooled cDNA. To assess the agreement between arrays, Bland-Altman statistical tests were applied. No significant differences (p > 0.50 in all cases) were observed when arrays performed on different occasions were analysed. Furthermore, changes in gene expression observed when arrays were performed on 2 separate occasions correlated significantly: DMOG-treated Caco-2 Spearman correlation co-efficient 0.42, p < 0.01, hypoxia-treated Caco-2 Spearman correlation co-efficient 0.29, p < 0.05, DMOG plus EGF-treated Caco-2 Spearman correlation co-efficient 0.49, p < 0.001.

### Analysis of protein expression

For the HIF-1α ELISA, cells were harvested and lysed in 50 mM TRIS, 300 mM NaCl, 3 mM EDTA, 1 mM MgCl_2_, 25 mM NaF, 20 mM β-glycerophosphate, 1% Triton-X, 10% glycerol and protease inhibitor cocktail P-8340 (Sigma, St Louis, MO, USA). Total protein was quantified by the Bicinchoninic assay (BCA) (Pierce, Rockford, USA). The HIF-1α Duoset IC (R&D Systems, Minneapolis, USA) was used to measure HIF-1α protein in total protein lysates. Results were analysed using Ascent Version 2.6 software (Thermo Fisher Scientific, Waltham, MA, USA).

Western blotting was performed using total protein lysates from cells harvested and lysed with urea buffer (8 M urea, 1% Sodium Dodecyl Sulphate, 1% glycerol and 10 mM Tris (pH6.8), 0.5 mM protease inhibitor cocktail (Sigma-Aldrich, Poole, UK), 1 mM dithiothreitol) for HIFs, or RIPA buffer (50 mM Tris pH 7.4, 150 mM NaCl, 1% Triton X, 0.1% SDS, 5 mM MgCl_2_, 50 mM NaF, 50 mM DTT, 2 mM orthovanadate, 5 mg/mL sodium deoxycholate, 10 mM sodium pyrophosphate, 25 mM β-glycerophosphate, 2 mM EDTA, 2 mM PMSF and protease inhibitor cocktail P-8340 (Sigma, St Louis, MO, USA) for signalling studies. Samples were resolved on SDS-polyacrylamide gels, where a 3-8% Tris-Acetate NuPAGE® Novex gel (Invitrogen, Carlsbad, CA, USA) was used for EGFR signalling studies, and a 4-12% Bis-Tris NuPAGE® Novex gel (Invitrogen, Carlsbad, CA, USA) was used for signalling and HIF-α protein studies. Rabbit anti-human phospho EGFR (Tyr 1068), phospho EGFR (Tyr 845), phospho p38 MAP Kinase (Thr 180/Tyr 182), phospho p44/42 MAP Kinase (Thr 202/Tyr 204), phospho-Akt (Ser 473), total EGFR, total p38 MAPK and total p44/42 MAPK were from Cell Signaling Technology (Danvers, MA, USA). Mouse anti-human HIF-1α and HIF-2α (EPAS) were from Becton Dickinson (Franklin Lakes, NJ, USA) and Santa Cruz Biotechnology (Santa Cruz, CA, USA) respectively. Secondary anti-rabbit and mouse HRP-conjugated antibodies were from DakoCytomation (Glostrup, Denmark). Whole cell lysate of EGF-treated A431 epithelial carcinoma cells used as positive control was from Santa Cruz Biotechnology (Santa Cruz, CA, USA). Densitometry was performed using Phoretix 1D analysis software (TotalLab Ltd, Newcastle upon Tyne, UK).

### Statistical analyses

Statistical significance was evaluated with 1-way ANOVA with Dunnett’s post-hoc test to compare selected groups of data. The ΔC_t_ values were used to determine the statistical significance of differences between groups for PCR-based studies. 2-way ANOVA with Bonferroni correction was used to compare selected groups of data with respect to time.

## Results

### HIF-dependent induction of angiogenic genes in Caco-2 cells in response to hypoxia and the hypoxia mimetic DMOG

**Figure 1:**
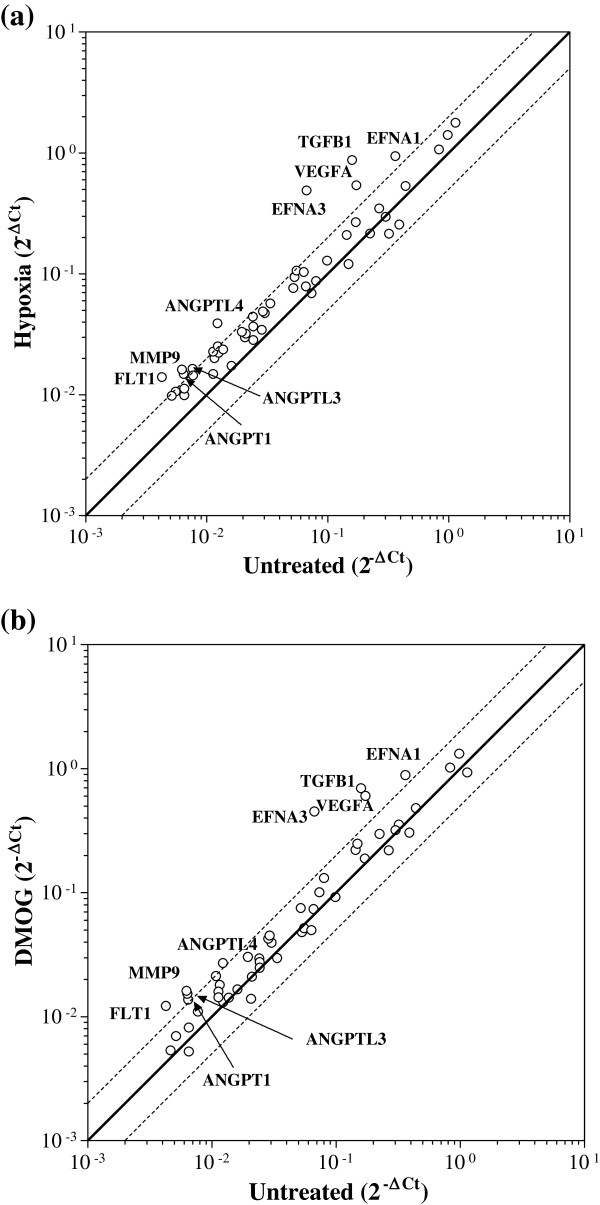
**Scatter plot of PCR angiogenesis array analysis of Caco-2 cells exposed to hypoxia and the hypoxia mimetic DMOG.** Caco-2 cells were exposed to either **(a)** hypoxia (1% O_2_) or **(b)** DMOG (1 mM) for 24 hours. Scatter plot graphs are 2^-ΔCt^ values for genes expressed by Caco-2 and normalised against HKG ActB (β-actin), 18S rRNA, HPRT1 (hypoxanthine phosphoribosyltransferase 1) and RPL13A (60S ribosomal protein L13a). Solid lines show no change, dashed lines show ≥2-fold increase and decrease *versus* untreated cells. Genes whose expression in both treated and untreated samples was below detection limits of the array are not included. Only genes whose expression changed ≥2-fold are annotated. Data are from a representative array performed in duplicate using cDNA pooled from 3 different replicate experiments.

Since hypoxia is likely to be a key stimulus for angiogenesis in CRC, we first investigated the angiogenic gene profile of Caco-2 cells exposed to either hypoxia or the hypoxia mimetic DMOG. Figure 1 and Table 1 illustrate the Human Angiogenesis RT^2^ Profiler™ PCR array data as scatter plots, and show that 9 pro-angiogenic genes were significantly changed by a factor of at least 2.0-fold in response to either hypoxia (Figure 1a) or DMOG (Figure 1b), including VEGF-A, known to be highly regulated by hypoxia in various cell types (fold increase 3.1 and 3.4 in response to hypoxia and DMOG respectively). Furthermore, 8 hypoxia-regulated genes were identified for the first time in Caco-2, namely angiopoietin (ANGPT) 1 (fold increase 2.3 and 2.1 in response to hypoxia and DMOG respectively), ANGPTL3 (fold increase 2.1 and 2.4), ANGPTL4 (fold increase 3.1 and 3.9), ephrin (EFN) A1 (fold increase 2.6 and 2.4), EFNA3 (fold increase 7.2 and 6.6), VEGF receptor FLT1 (fold increase 3.1 and 2.8), matrix metalloprotease (MMP) 9 (fold increase 2.6 and 2.6) and TGFβ1 (fold increase 5.4 and 4.3). None of the genes were downregulated in response to treatment. A significant correlation was observed between the fold-changes in gene expression observed in hypoxia- *versus* DMOG-treated Caco-2 cells (Spearman correlation co-efficient 0.50, p < 0.001; not shown), highlighting the high degree of concordance between hypoxia- and DMOG-mediated responses in Caco-2 CRC cells.

**Table 1:** Genes included on the angiogenesis PCR array and expressed by Caco-2 cells

**Symbol**	**Gene name**	**Description**	**Stimulus**			
			**Hypoxia**	**DMOG**	**EGF**	**EGF + DMOG**
AKT1	PKB/PRKBA	V-akt murine thymoma viral oncogene homolog 1	−1.1	1.3	1.2	1.1
ANGPT1	AGP1/AGPT	Angiopoietin 1	**2.3**	**2.1**	1.6	**2.3**
ANGPTL3	ANGPT5	Angiopoietin-like 3	**2.1**	**2.4**	1.3	**2.4**
ANGPTL4	ANGPTL2/ARP4	Angiopoietin-like 4	**3.1**	**3.9**	1.4	**5.8**
ANPEP	CD13/LAP1	Alanyl (membrane) aminopeptidase (aminopeptidase N, aminopeptidase M, microsomal aminopeptidase, CD13, p150)	1.5	1.3	−1.4	1.4
CCL11	SCYA11	Chemokine (C-C motif) ligand 11	1.9	1.1	1.5	**3.5**
CCL2	GDCF-2/GDCF-2 HC11	Chemokine (C-C motif) ligand 2	1.4	−1.5	1.6	1.4
COL18A1	KNO	Collagen, type XVIII, alpha 1	1.8	1.2	1.0	1.3
COL4A3	TUMSTATIN	Collagen, type IV, alpha 3 (Goodpasture antigen)	1.7	−1.1	1.3	**2.2**
EDG1	CHEDG1/D1S3362	Endothelial differentiation, sphingolipid G-protein-coupled receptor 1, SIPR1	1.7	1.5	1.7	**3.0**
EFNA1	B61/ECKLG	Ephrin-A1	**2.6**	**2.4**	1.0	**2.0**
EFNA3	EFL2/EPLG3	Ephrin-A3	**7.2**	**6.6**	1.3	**4.9**
EFNB2	EPLG5/HTKL	Ephrin-B2	1.4	1.3	1.3	1.6
ENG	CD105/END	Endoglin	1.1	1.1	1.5	1.2
EPHB4	HTK/MYK1	EPH receptor B4	1.2	1.0	1.5	1.3
EREG	ER	Epiregulin	1.2	1.1	1.2	−1.9
FGFR3	ACH/CEK2	Fibroblast growth factor receptor 3	1.4	1.5	1.2	1.4
FLT1	FLT/VEGFR1	Fms-related tyrosine kinase 1	**3.1**	**2.8**	1.4	**2.6**
HIF1A	HIF-1alpha	Hypoxia-inducible factor 1, alpha	−1.5	1.0	1.0	1.0
HPSE	HPA/HPR1	Heparanase	1.7	1.9	1.1	1.8
ID1	ID	Inhibitor of DNA binding 1, dominant negative helix-loop-helix protein	−1.3	1.6	1.7	1.9
ID3	HEIR-1	Inhibitor of DNA binding 3, dominant negative helix-loop-helix protein	1.5	−1.0	1.4	**2.5**
IGF1	IGFI	Insulin-like growth factor 1	1.9	1.4	1.2	1.6
IL6	BSF2/HGF	Interleukin 6 (interferon, beta 2)	1.9	1.3	1.5	1.7
IL-8	3-10C/AMCF-I	Interleukin 8	1.9	−1.1	1.2	**2.4**
ITGAV	CD51/MSK8	Integrin, alpha V (CD51)	−1.1	1.4	1.9	1.9
ITGB3	CD61/GP3A	Integrin, beta 3 (platelet glycoprotein IIIa, antigen CD61)	1.3	−1.1	−1.2	**2.4**
JAG1	AGS/AHD	Jagged 1 (Alagille syndrome)	1.1	1.6	1.6	**2.7**
KDR	FLK1/VEGFR	Kinase insert domain receptor	1.7	−1.2	−1.2	**2.3**
LAMA5	KIAA1907	Laminin, alpha 5	1.6	1.2	1.0	1.0
MMP2	CLG4/CLG4A	Matrix metallopeptidase 2 (gelatinase A, 72kDa gelatinase, 72kDa type IV collagenase)	1.7	1.5	−1.3	1.6
MMP9	CLG4B/GELB	Matrix metallopeptidase 9 (gelatinase B, 92kDa gelatinase, 92kDa type IV collagenase)	**2.6**	**2.6**	1.7	**2.4**
NOTCH4	INT3/NOTCH3	Notch homolog 4 (Drosophila)	1.5	−1.3	1.0	**2.0**
NRP1	DKFZp686A03134/DKFZp781F1414	Neuropilin 1	−1.6	−1.3	−1.5	−1.1
NRP2	NP2/NPN2	Neuropilin 2	−1.0	1.0	1.1	1.5
PDGFA	PDGF-A/PDGF1	Platelet-derived growth factor alpha polypeptide	1.2	1.5	1.2	−1.2
PECAM1	CD31/PECAM-1	Platelet/endothelial cell adhesion molecule (CD31 antigen)	1.61	−1.3	1.0	1.7
PLAU	ATF/UPA	Plasminogen activator, urokinase	1.4	1.4	1.4	1.7
PLXDC1	TEM3/TEM7	Plexin domain containing 1	1.9	1.0	1.7	1.8
SERPINF1	EPC-1/PEDF	Serpin peptidase inhibitor, clade F (pigment epithelium derived factor)	1.5	1.1	1.2	1.5
SPHK1	SPHK	Sphingosine kinase 1	1.5	1.0	1.6	**2.0**
TGFA	TFGA	Transforming growth factor, alpha	1.9	1.4	1.5	**2.3**
TGFB1	CED/DPD1	Transforming growth factor, beta 1	**5.4**	**4.3**	1.4	**4.6**
TGFB2	TGF-beta2	Transforming growth factor, beta 2	1.7	1.0	1.0	1.8
TGFBR1	ACVRLK4/ALK-5	Transforming growth factor, beta receptor I (activin A receptor type II-like kinase, 53 kDa)	1.6	1.5	1.3	1.6
THBS1	THBS/TSP	Thrombospondin 1	1.3	1.2	1.0	1.0
THBS2	TSP2	Thrombospondin 2	1.1	1.0	−1.2	−1.1
TIMP1	CLGI/EPA	TIMP metallopeptidase inhibitor 1	1.3	−1.2	−1.1	1.1
TIMP2	CSC-21K	TIMP metallopeptidase inhibitor 2	1.5	−1.2	−1.3	−1.1
TNFAIP2	B94	Tumour necrosis factor, alpha-induced protein 2	1.7	1.2	−1.1	1.4
VEGF	VEGFA/VPF	Vascular endothelial growth factor	**3.1**	**3.4**	1.0	**3.1**

The Human Angiogenesis RT^2^ Profiler™ PCR Array was used to screen cDNA from Caco-2 cells exposed for 24 hours to either 1% O_2_, DMOG (1 mM), EGF (10 ng/ml) or a combination of EGF plus DMOG. Data were compared to HKG: ActB (β-actin), 18S rRNA, HPRT1 (hypoxanthine phosphoribosyltransferase 1) and RPL13A (60S ribosomal protein L13a), and are fold change *versus* untreated cells. Changes ≥2-fold are shown in bold. Data are from a representative array performed in duplicate using cDNA pooled from 3 different replicate experiments.

**Figure 2:**
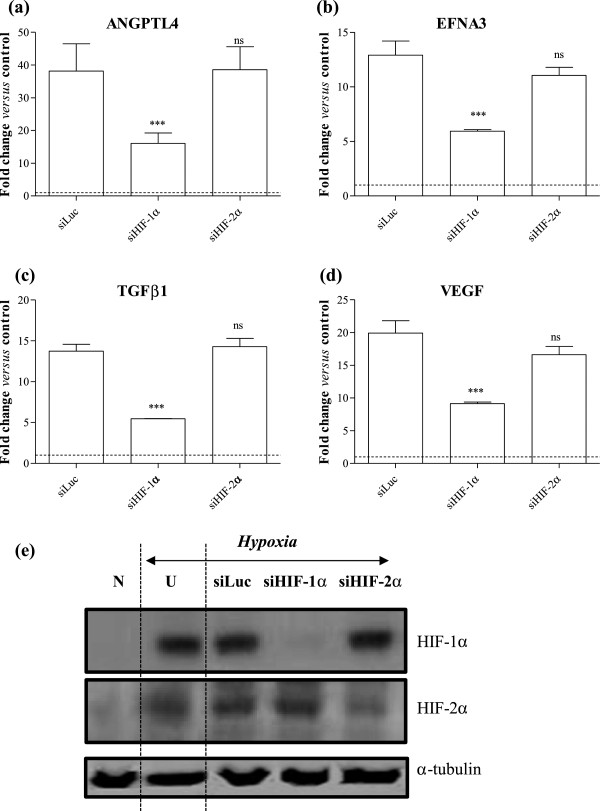
**Angiogenic gene expression in Caco-2 cells exposed to hypoxia is HIF-1 dependent.** Caco-2 cells were transfected with siRNA targeting luciferase (siLuc), HIF-1a (siHIF-1a) or HIF-2a (siHIF-2a), and subsequently exposed to hypoxia (1% O_2_) for 24 hours. Changes in **(a)** ANGPTL4, **(b)** EFNA3, **(c)** TGFβ1 and **(d)** VEGF mRNA levels were determined by Q-PCR using the 2^-ΔΔCt^ method and are expressed relative to HKG 18S. Data are mean ± SEM from 3 representative experiments, and were analysed by 1-way ANOVA of ΔC_t_ values *versus* siLuc: ns = not significant, *** p < 0.001. Dashed line shows response of cells exposed to normoxia (21% O_2_). **(e)** Western blots, demonstrating specific HIF knockdown at the protein level, with α-tubulin shown as loading control. N = normoxia, U = untransfected cells exposed to hypoxia.

**Figure 3:**
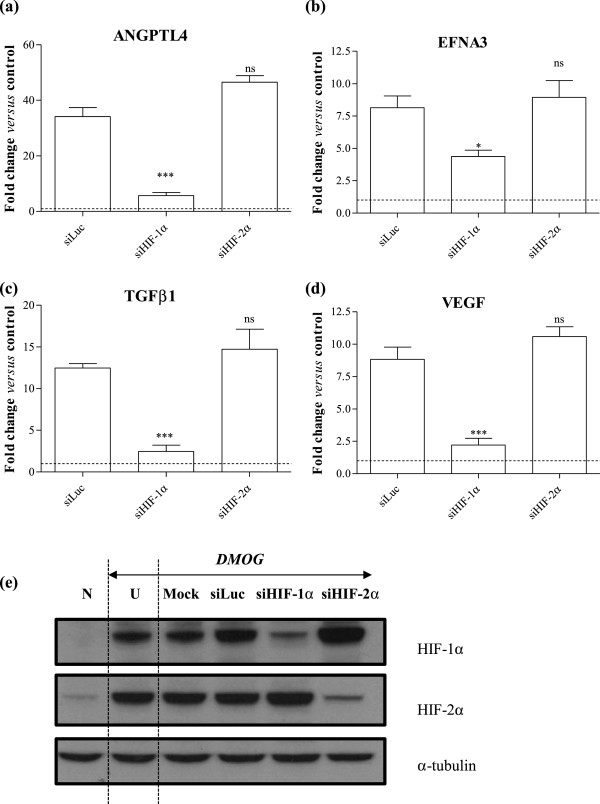
**Angiogenic gene expression in Caco-2 cells exposed to DMOG is HIF-1 dependent.** Caco-2 cells were transfected with siRNA targeting luciferase (siLuc), HIF-1α (siHIF-1α) or HIF-2α (siHIF-2α), or with lipofectamine alone (Mock), and subsequently exposed to DMOG (1mM) for 24 hours. Changes in **(a)** ANGPTL4, **(b)** EFNA3, **(c)** TGFβ1 and **(d)** VEGF mRNA levels were determined by Q-PCR using the 2^-ΔΔCt^ method and are expressed relative to HKG 18S. Data are mean ± SEM from 3 representative experiments, and were analysed by 1-way ANOVA of ΔC_t_ values *versus* siLuc: ns = not significant, * p < 0.05, *** p < 0.001. Dashed line shows response of unstimulated cells. **(e)** Western blots, demonstrating HIF knockdown at the protein level, with α-tubulin shown as loading control. N = normoxia, U = untransfected cells exposed to DMOG.

The genes whose expression changed the most dramatically in response to hypoxia and DMOG were ANGPTL4, EFNA3, TGFβ1 and VEGF. To determine their requirement for HIF isoforms, a small interfering (si) RNA approach was used. Specific knockdown of HIF-1α and HIF-2α, which we have previously demonstrated in other cell types to markedly reduce HIF mRNA and protein [38,39], was confirmed in Caco-2 at the mRNA level in both DMOG- and hypoxia-stimulated cells, with 81% and 85% knockdown of HIF-1α mRNA in the presence of siRNA against HIF-1α (compared with siLuc-transfected Caco-2 cells), and 93% and 86% knockdown of HIF-2α mRNA in the presence of siRNA against HIF-2α (data not shown). There was no inhibitory effect of siHIF-1α on HIF-2α, and *vice versa* (data not shown). Specific knockdown of HIF-1α and HIF-2α was also observed at the protein level in cells exposed to hypoxia (Figure 2e) and DMOG (Figure 3e).

Expression of ANGPTL4 was dependent on HIF-1α in Caco-2 cells stimulated with either hypoxia or DMOG (Figures 2a and 3a), with reductions of 83% (relative to siLuc-transfected cells; p < 0.001) and 60% (p < 0.001) respectively. In contrast, knockdown of HIF-2α was without effect. Comparable data were observed for the other genes in cells exposed to hypoxia, with knockdown of HIF-1α, but not of HIF-2α, having a significant inhibitory effect. Thus for EFNA3, reductions of 54% (p < 0.001; Figure 2b) and 43% (p < 0.05; Figure 3b) were observed in response to hypoxia and DMOG respectively in the presence of siHIF-1α. For TGFβ1, reductions of 60% (p < 0.001; Figure 2c) and 80% (p < 0.001; Figure 3c) were observed in response to hypoxia and DMOG respectively. Finally, in the case of VEGF, HIF-1α knockdown resulted in reductions of 54% (p < 0.001; Figure 2d) and 75% (p < 0.001; Figure 3d) in response to hypoxia and DMOG respectively. These findings suggest that HIF-1, but not HIF-2, mediates the induction of angiogenic genes in CRC cells downstream of HIF activation in response to ether hypoxia or the hypoxia mimetic DMOG.

### Analysis of Caco-2 responses to EGF alone and in combination with the hypoxia mimetic DMOG

**Figure 4:**
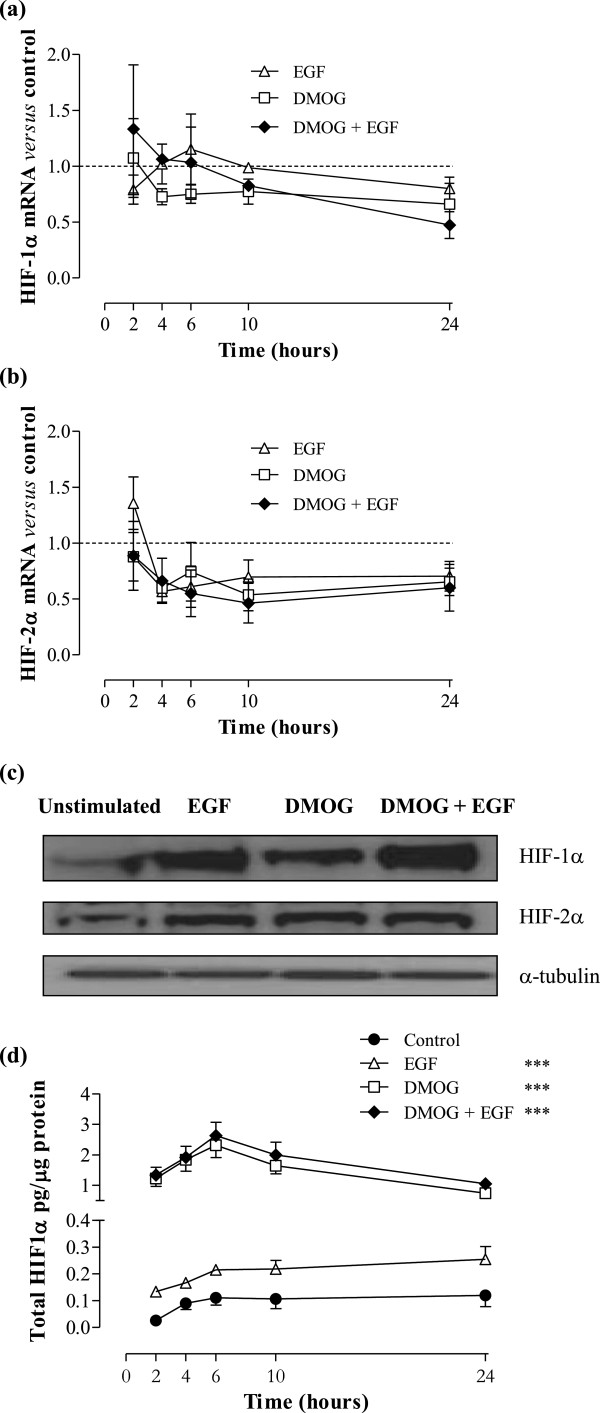
**HIF-α in Caco-2 cells exposed to EGF and/or DMOG.** Caco-2 cells were stimulated with 20ng/mL EGF and/or 1mM DMOG for the time periods indicated. Fold change in **(a)** HIF-1α and **(b)** HIF-2α mRNA levels were determined by Q-PCR using the 2^-ΔΔCt^ method and are expressed relative to HKG 18S. Data are mean ± SEM from 2 representative experiments. **(c)** HIF-1α and HIF-2α protein was measured by Western blotting in Caco-2 cells stimulated with EGF and/or DMOG for 24 hours. α-tubulin is shown as a loading control. **(d)** HIF-1α protein was measured by ELISA. Data are mean ± SEM from 3 representative experiments, and were analyzed by 2-way ANOVA versus unstimulated cells: *** p < 0.001.

Since we established that angiogenic gene induction was HIF dependent in Caco-2 cells, we next investigated the effect of EGF, alone or in combination with the hypoxia mimetic agent DMOG, on activation of the HIF pathway in Caco-2 cells. HIF-1α (Figure 4a) and HIF-2α (Figure 4b) mRNA decreased modestly following stimulation with either EGF, DMOG or a combination of both EGF and DMOG stimulation, but these differences in level of mRNA across all three groups over a period of 24 hours were not statistically significant. In contrast, Western blot analysis demonstrated a consistent up-regulation of both HIF-1α and HIF-2α protein following DMOG or EGF stimulation alone and in combination (Figure 4c). Analysis using ELISA for HIF-1α confirmed the observation that EGF resulted in a modest but statistically significant increase in HIF-α protein levels, but addition of EGF to DMOG did not further increase the HIF-1α response relative to that seen with DMOG alone. After 24 hours, HIF-1α protein levels were equivalent to 0.12 ± 0.04 pg/μg total protein in unstimulated Caco-2 compared with 0.25 ± 0.05 pg/μg total protein in EGF-treated cells (p < 0.05 *versus* untreated cells), compared to 0.74 ± 0.03 pg/μg total protein (p < 0.001) and 0.88 ± 0.18 pg/μg total protein (p < 0.001) in cells exposed to DMOG alone or DMOG in combination with EGF (Figure 4d).

**Figure 5:**
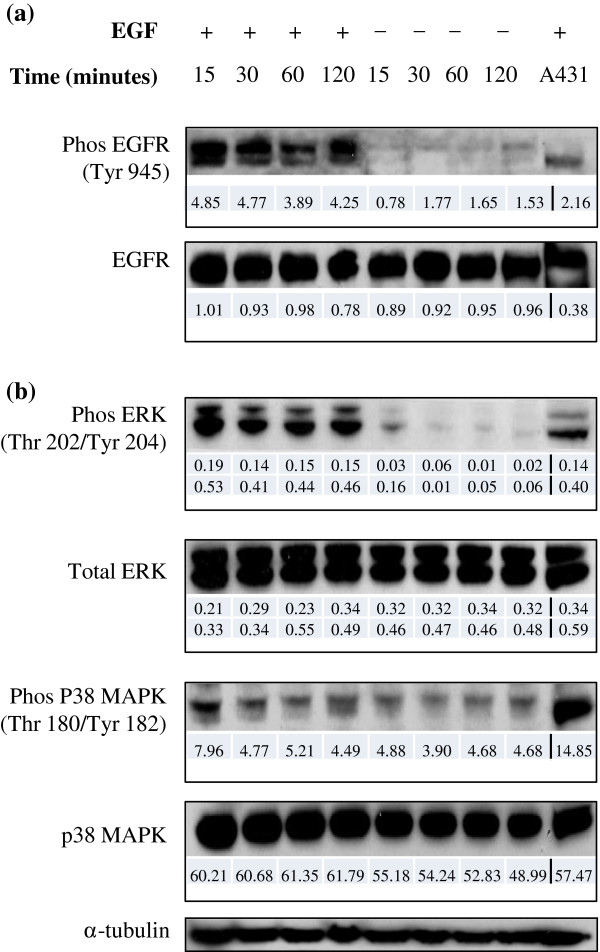
**EGF receptor is autophosphorylated in Caco-2 and activates downstream signalling pathways.** Caco-2 cells were stimulated with 20 ng/mL EGF for the time periods indicated. Western blotting for **(a)** phosphorylated EGFR or total EGFR and **(b)** antibodies recognising signalling enzymes is shown. Cell lysate of EGF-treated A431 cells was used as positive control. α-tubulin is shown as a loading control. Densitometry was performed using Phoretix 1D analysis software against α-tubulin (for ERK, data for p42 and p44 are shown).

To investigate whether Caco-2 cells can respond to EGF stimulation to activate other signalling pathways, cells were exposed to EGF for different periods of time, or left unstimulated. Figure 5a illustrates that a protein band corresponding to phospho-EGFR was observed following EGF stimulation, with marked phosphorylation of Tyr 945 in the intracellular signalling portion of the receptor. The peak of receptor activation was seen 15–30 minutes following stimulation, and progressively declined over the course of 60–120 minutes. Modest autophosphorylation of Tyr 1068 following EGF stimulation was also observed (data not shown).

Downstream signalling pathways known to play a role in Caco-2 cells [40,41] were investigated as potential signal transducers involved in initiating various intracellular activities resulting from EGF-induced EGFR autophosphorylation. Figure 5b confirms markedly higher expression of phosphorylated p44 MAPK (ERK1) at Thr 202 and p42 MAPK (ERK2) at Tyr 204 in EGF-stimulated *versus* control cells, which was maintained even 2 hours after stimulation. The presence of anti-phospho-p38 MAPK protein bands in both stimulated and unstimulated cells suggests basal activation of p38 MAPK in Caco-2, which is not further increased by EGF (although a very modest increase of less than 2-fold was observed 15 minutes after EGF addition). Akt phosphorylation in Caco-2 cells was analysed and found to be constitutively activated in Caco-2 cells (data not shown).

### Angiogenic gene profiling of Caco-2 cells following EGFR activation

**Figure 6:**
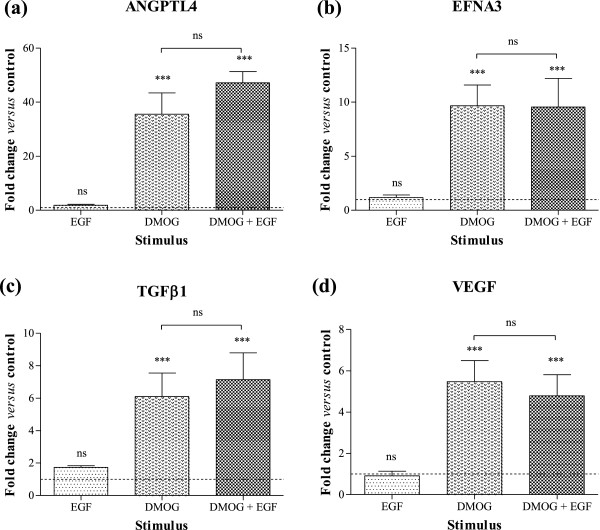
**Angiogenic gene expression in Caco-2 cells exposed to EGF and/or DMOG.** Caco-2 cells were stimulated with 20 ng/mL EGF and/or 1 mM DMOG for 24 hours. Changes in **(a)** ANGPTL4, **(b)** EFNA3, **(c)** TGFβ1 and **(d)** VEGF mRNA levels were determined by Q-PCR using the 2^-ΔΔCt^ method and are expressed relative to HKG 18S. Data are mean ± SEM from 3 representative experiments, and were analysed by 1-way ANOVA of ΔC_t_ values *versus* normoxia (unless otherwise indicated): ns = not significant, *** p < 0.001. Dashed line shows response of unstimulated cells.

The above cell signalling studies clearly demonstrate that EGF is capable of activating downstream signalling in Caco-2 cells, inducing rapid phosphorylation of tyrosine residues in EGFR, activation of ERK1/2 and stabilisation of HIF proteins. However, in spite of the observed changes, and in particular despite stabilisation of HIF-1α, expression of the 4 angiogenic HIF-1 target genes, namely ANGPTL4 (Figure 6a), EFNA3 (Figure 6b), TGFβ1 (Figure 6c) and VEGF (Figure 6d), was unaffected by addition of EGF alone. Furthermore, responses induced by DMOG alone were not further altered by addition of EGF (p > 0.05 versus DMOG alone) specifically for these 4 angiogenic genes.

**Figure 7:**
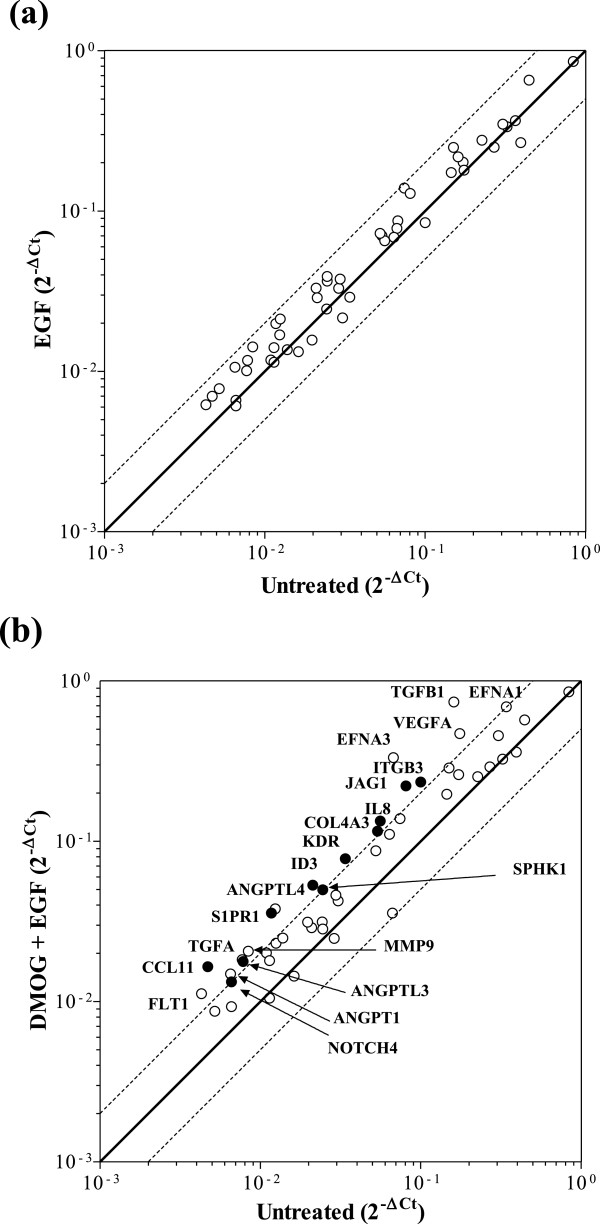
**Scatter plot PCR array analysis of Caco-2 cells exposed to EGF alone or in combination with DMOG.** Caco-2 cells were stimulated with 20 ng/mL EGF for 24 hours alone **(a)** or in combination with 1mM DMOG **(b)**. Scatter plot graphs are 2^-ΔCt^ values for genes expressed by Caco-2 and normalised against HKG ActB (β-actin), 18S rRNA, HPRT1 (hypoxanthine phosphoribosyltransferase 1) and RPL13A (60S ribosomal protein L13a). Solid lines show no change, dashed lines show ≥2-fold increase and decrease *versus* untreated. Genes whose expression in both treated and untreated samples was below detection limits of the array are not included. Only genes whose expression changed ≥2-fold are annotated, with annotated open circles representing the 9 genes also changed in response to hypoxia and DMOG, and annotated closed circles representing the 11 additional genes uniquely changed only in response to DMOG plus EGF. Data are from a representative array performed in duplicate using cDNA pooled from 3 different replicate experiments.

The Human Angiogenesis RT^2^ Profiler™ PCR Array was used to examine the expression of a panel 84 established angiogenic genes in cells exposed to either EGF alone or in combination with DMOG. None of the genes which were detected on the array demonstrated significant change in expression (either upregulation or downregulation) following EGFR activation (Figure 7a and Table 1). Combined DMOG and EGF did not further induce expression of the 9 genes previously shown to be upregulated by DMOG alone or hypoxia alone (ANGPT1, ANGPTL3, ANGPTL4, EFNA1, EFNA3, FLT1, MMP9, TGFβ1 and VEGF, Figure 7b and Table 1). Nevertheless, the combined stimuli induced a unique profile of 11 additional angiogenic genes which were not altered by either hypoxia alone, DMOG alone or EGF alone. Specifically, expression of chemokines CCL11 (eotaxin-1; 3.5-fold increase) and IL8 (2.4-fold), together with EDG1 (endothelial differentiation gene 1 or sphingolipid G-protein-coupled receptor 1; 3.0-fold increase), DNA-binding protein inhibitor ID3 (2.5-fold increase), Jagged 1 (known also as CD339; 2.7-fold increase), VEGF receptor KDR (2.3-fold increase), NOTCH4 (2.0-fold increase), SPHK1 (sphingosine kinase 1; 2.0-fold increase) and TGFα (2.3-fold increase) was altered in response to EGF plus DMOG (Figure 7b and Table 1). Furthermore, expression of COL4A3 was also increased (2.2-fold) in Caco-2 exposed to the combination of EGF plus DMOG, as were levels of integrin β3 chain (2.4-fold). These findings demonstrate that there are 2 unique gene signatures in Caco-2 cells, namely a set of 9 genes affected by hypoxia/DMOG alone, and a further set of 11 genes induced only by combined EGF and DMOG stimulation.

## Discussion

CRC is the third most common cancer worldwide, and in the European Union alone, the lifetime estimated risk of developing the disease is 6%. Over the last 30 years, advances in diagnostic tools and a consensus towards internationally standardised staging criteria of the condition, together with combined multimodal treatment strategies, have contributed to substantial improvement in 5 year survival rates for patients with CRC, from 22% to 50% [42]. Crucially, recent advances in understanding molecular mechanisms driving tumours have increased our understanding of the mechanisms underlying the benefits of new treatment agents which selectively target abnormal pathways confined to tumours, allowing improvements in the prognosis of patients with advanced CRC and development of new therapeutic modalities.

Deciphering the complex biological mechanisms underlying tumour angiogenesis has been a major focus of research, as the growth of solid tumours is restricted to 2-3 mm^3^ in size without neo-vascularisation [43]. Hypoxia, a feature common to most solid tumours, has been established as a promoter of angiogenesis by modulating expression of several mediators, particularly VEGF, cell adhesion molecules and surface receptors. However, hypoxia-regulated candidate genes specifically relevant to CRC angiogenesis have not been examined in detail. Caco-2 CRC cells are an adherent cell line isolated from a patient with colorectal adenocarcinoma. Their capacity to differentiate into a polarised monolayer of mature enterocyte-like cells on reaching confluence, which has led to their adoption as a standard model for *in vitro* studies of enteric drug absorption and transport [44], and their widespread used as an *in vitro* model of CRC [36,41,45,46]. In common with approximately 50% of colorectal tumours, Caco-2 cells have a mutant p53 oncogene, which is known to be associated with increased VEGF production [47]. Caco-2 cells contain the wild-type of two other oncogenes, K-ras and BRAF [48,49], mutations of which are present in 45% and 15% of colorectal tumours respectively [49,50]. Furthermore, Caco-2 express receptors for EGF and release VEGF in response to number of stimuli including hypoxia and K-ras [14,51-53]. Inappropriate mucin gene expression is also related to CRC development, invasiveness and prognosis, and mucin-5AC, which is expressed in large amounts in Caco-2 cells, has been observed in the early stages of the colorectal adenoma-carcinoma sequence [49,54]. In addition, Claudin-2, a unique member of the claudin family of transmembrane proteins which is significantly increased in CRC and correlates with cancer progression and tumour growth, is regulated in Caco-2 via EGF [55]. Caco-2 tumourigenicity has been demonstrated by the development of moderately-well differentiated adenocarcinoma *in vivo* following inoculation into mice [56]. Use of Caco-2 cells thus allows elucidation of mechanisms of disease pathogenesis, including angiogenesis [57,58], with pathway-based analysis likely to yield valuable information at the molecular level that would contribute to our understanding of the development of CRC.

The present study identified VEGF-A, known to be regulated by hypoxia in other cell types, as a hypoxia-responsive gene in CRC cells, together with 8 additional hypoxia-regulated genes namely ANGPT1, ANGPTL3, ANGPTL4, EFNA1, EFNA3, VEGF receptor FLT1, MMP9 and TGFβ1. An identical angiogenic gene signature relevant to CRC was elicited following treatment of Caco-2 with the pan-specific HIF hydroxylase inhibitor and HIF activator DMOG. Genes with the highest change in expression following hypoxia or DMOG stimulation, namely ANGPTL4, EFNA3, TGFβ1 and VEGF, were selected for studies using RNA knockdown. Previous studies have demonstrated that hypoxic induction of VEGF in Caco-2 cells was in part due to HIF-1α, but this study did not detect significant levels of HIF-2α [14]. A study by Zgouras *et al.* showing that HIF-1α regulates butyrate-induced normoxic VEGF expression in Caco-2 cells did not investigate the possible involvement of HIF-2α [57], and while studies have linked HIF-1α expression with apoptosis in Caco-2, none examined the role of HIF-2α [17,59]. In our study, the increase in ANGPTL4, EFNA3, TGFβ1 and VEGF expression by hypoxia was significantly inhibited following knockdown of HIF-1α, with little or no contribution of HIF-2α. Thus, we have established a unique set of angiogenic genes which were hypoxia-regulated in CRC Caco-2 cells, and confirmed an identical expression profile with DMOG stimulation, as well as the dependence of angiogenic responses on HIF-1 by RNA knockdown studies.

In addition to the oxygen-dependent regulation of HIF-α by hypoxia and hypoxia mimetics such as DMOG, signalling by growth factors including EGFR activation has been shown to induce HIF-1α expression in other cell types under normoxic conditions [60]. The key role of EGF/EGFR in CRC has been demonstrated by the successful development of EGFR-targeted therapies cetuximab and panitumumab. Our study confirmed that EGFR autophosphorylation is associated with HIF-1α and HIF-2α protein stabilisation under normoxia in Caco-2 cells. Unlike the effect of hypoxia on protein stability due to the inactivity of oxygen-dependent HIF hydroxylases, the observed increase in HIF-α protein is most probably attributed to post-transcriptional responses, such as increased stability or post-translational modifications, since mRNA levels of HIF-1α and HIF-2α were not increased by EGF. A study on breast cancer cells where HER2 signalling specifically induced HIF-1α protein expression without affecting HIF-1α mRNA showed the response was dependent upon activation of the PI3K/Akt/FRAP thus increasing rate of protein synthesis [31]. Other studies have also reported increased HIF-1α translation mediated through PI3K/Akt [33,61]. In order to investigate the involvement of a similar signalling pathway, we examined activation of EGFR, ERK and p38 MAPK and Akt. Our study on Caco-2 cells illustrated selective activation of MAPK ERK1/2 signalling, in contrast to PI3K/Akt and P38 MAPK which remained constitutively active irrespective of exogenous EGFR stimulation.

Since EGFR activation led to HIF upregulation in Caco-2 cells, a response analogous to that observed with hypoxia or DMOG, we predicted that EGFR-induced angiogenic gene profile would parallel that induced by hypoxia or DMOG. Such findings would lend further impetus towards developing novel anti-EGFR agents such as the monoclonal antibodies cetuximab and panitumumab [26,28]. The next part of our study therefore aimed to decipher the global involvement of known angiogenic genes in modulating the tumour microenvironment. Unexpectedly, our data showed that none of the 84 angiogenic genes were affected by EGFR activation, in spite of induction of downstream ERK MAPK signalling and stabilisation of HIF-α. The absence of effect of EGF alone was also validated by Q-PCR for ANGPTL4, EFNA3, TGFβ1 and VEGF, genes which demonstrated significant upregulation in a HIF-1-dependent manner following exposure of Caco-2 to DMOG or hypoxia. However, both EGFR over-activation and hypoxia typically co-exist within the tumour microenvironment and both may impact upon the differential modulation of angiogenic responses induced by either stimulus. We therefore examined the effect of simultaneous stimulation of Caco-2 CRC cells using EGF and the HIF activator DMOG. Our data demonstrated that the previously established hypoxia-regulated angiogenic genes (ANGPT1, ANGPTL3, ANGPTL4, EFNA1, EFNA3, FLT1, MMP9, TGFβ1 and VEGF) were not further affected by addition of EGF. Importantly, we have instead identified an additional sub-set of genes which were only expressed following combined EGF and DMOG, and not with either EGF alone or DMOG/hypoxia alone. The unique profile of 11 additional angiogenic genes which were only expressed with combined EGF and DMOG includes chemokines CCL11 (eotaxin-1) and IL8, EDG1 (endothelial differentiation gene 1 or sphingolipid G-protein-coupled receptor 1), DNA-binding protein inhibitor ID3, Jagged 1 (JAG1 known also as CD339), VEGF receptor KDR, NOTCH4, SPHK1 (sphingosine kinase 1, which extracellularly acts as a ligand for EDG1) and TGFα. Furthermore, expression of COL4A3 (tumstatin, an angiogenesis inhibitor which is a cleavage fragment of collagen IV α3 NC1 domain) was also increased in Caco-2 exposed to the combination of EGF plus DMOG, as were levels of integrin β3 chain, which together with αV integrin binds tumstatin via an RGD-independent mechanism. As both EGFR [20] and hypoxia [6] are inducers of angiogenesis, these results suggest a novel and previously unreported synergistic relationship which culminates in a downstream response that supersedes the angiogenic effect exerted by either of the stimuli in isolation. This synergistic effect may be explained by the positive influence of activated ERK MAPK downstream of EGFR on the activity of HIF complexes by enhancing recruitment of p300/CREB-binding protein (CBP), thus completing the formation of functionally active transcription complexes to transactivate hypoxia response elements of select genes [62]. However it remains unclear why a similar response is not elicited in Caco-2 following EGFR activation alone, given that HIF expression was significantly upregulated (paralleling that following DMOG treatment) and downstream ERK MAPK signalling was activated. It is conceivable that despite activated EGFR increasing expression of HIF, this transcription factor is functionally inactive due to the activity of HIF hydroxylase enzymes such as factor inhibiting HIF-1 (FIH-1), which interferes with the ability of HIF to initiate transcription. Under normoxic conditions, hydroxylation of the asparagine residue 803 in the carboxyl-terminal transcriptional activation domain of HIF abrogates interactions with the transcriptional co-activators p300 and CBP [63]. Translation of results from our study to the clinical setting suggests that inhibition of angiogenesis with EGFR antagonists may be better targeted at select tumours which are particularly hypoxic.

The precise roles of ANGPTL4, EFNA3 and TGFβ1, and the 11 unique genes induced by EGF plus DMOG which are not induced by DMOG or hypoxia alone, in regulating CRC angiogenesis remain unknown. ANGPTL4 is a member of a family of seven molecules bearing structural homology to angiopoietins [64], and appears to mediate both pro- and anti-angiogenic effects, with the eventual outcome determined by cell-specific contexts and interactions with other angiogenic factors [65-67]. Of relevance, a recent study has reported that expression of ANGPTL4 correlates with the depth of tumour invasion, venous invasion and Duke’s classification in CRC [68]. EFNA3 was another novel gene identified as being upregulated by DMOG and hypoxia in Caco-2 cells. Ephrins and their cognate receptor tyrosine kinases regulate cell migration and adhesion, and thereby influence cell lineage, morphogenesis and organogenesis [69,70]. In adult life, ephrin upregulation, particularly of ephrin B, has been correlated to vascular invasion, blood vessel formation and sprouting by tumours, and soluble Eph A receptors have been shown to inhibit tumour angiogenesis [71]. The role of EFNA3 in CRC angiogenesis remains unproven, although ephrin and Eph receptor over-expression has been reported in a variety of human cancers including CRC [72,73]. TGFβ has a multifaceted homeostatic role in regulating cell growth and differentiation, angiogenesis, immune function and extracellular matrix formation [74]. Overexpression of TGFβ1 in primary CRC is a poor prognostic predictor and correlated with advanced stage of disease, increased risk of recurrence, shorter post-operative survival, particularly in early tumours and decreased overall survival [75,76]. Regulation of TGFβ1 expression by tissue oxygenation remains unstudied in CRC, although HIF-1α has been shown to increase TGFβ expression in prostate cancer cells [77]. Immunohistochemical studies have demonstrated a correlation between TGFβ and VEGF expression, where CRC tissues with the highest microvessel density expressed both growth factors [78].

Although the focus of the study was to investigate the angiogenic responses induced by EGFR, the receptor, being a member of the ErbB family of receptor tyrosine kinases, also has influence over numerous cellular processes by triggering multiple signalling cascades. EGFR signalling promotes DNA synthesis and cell cycle progression by recruiting downstream MAPK, STAT proteins, SRC family and Akt protein kinases, which can induce transcription of genes involved in cell growth, division, differentiation and survival [79-82]. Pre-clinical and clinical data show that aberrant EGFR and downstream signalling results in cellular transformation which can lead to sustained proliferation of abnormal malignant cells [82-84]. Furthermore, stimulation of EGFR pathways has been shown to promote tumour cell invasion, motility, adhesion and metastasis [85,86]. Despite the inability to demonstrate angiogenic gene responses following EGFR activation in our study, EGFR remains an important feature as preclinical and clinical studies have demonstrated efficacy of EGFR inhibitors in advanced CRC, particularly in combination with chemo- and radiotherapy [87,88].

## Conclusion

In summary, we have identified three novel HIF-1α-regulated angiogenic genes in Caco-2 cells, of which two, ANGPTL4 and TGFβ1, are associated with worse outcome in patients with CRC. In this regard, it is relevant that we have recently observed that primary cells isolated enzymatically from tumour resections obtained from patients with CRC also upregulate expression of VEGF, EFNA3, TGFβ1 and ANGPTL4 when exposed to hypoxia, supporting the relevance of studies using Caco-2 cells to understand the mechanisms underlying CRC progression and underlining the potential importance of these angiogenic genes in CRC [89-91]. We subsequently studied Caco-2 responses to EGF, the action of which is inhibited by successful CRC treatments, that is anti-EGFR antibodies cetuximab and panitumumab. However, despite our finding that EGFR autophosphorylation led to selective downstream activation of p42/p44MAPK and HIF protein stabilisation, this was not sufficient to induce angiogenic gene responses in CRC cells. In contrast, EGF synergised with the hypoxia mimetic DMOG to induce the expression of a unique subset of angiogenic genes. Our findings support a key role for tissue hypoxia in eliciting angiogenic gene responses in CRC cells, also in combination with EGF, and highlight the complex interrelationship between tumour hypoxia, EGF and angiogenesis in the pathogenesis of CRC.

## Abbreviations

ANGPT1: Angiopoietin 1; ANGPTL: Angiopoietin like; COL4A3: Tumstatin, cleavage fragment of collagen IV α3 NC1 domain; CRC: Colorectal cancer; DMOG: Dimethyloxalylglycine; EFN: Ephrin; EGF(R): Epidermal growth factor (receptor); FLT1: Vascular endothelial growth factor receptor 1; HER: Human epidermal receptor; HIF: Hypoxia inducible factor; HKG: House keeping gene; IL8: Interleukin 8; MMP: Matrixmetalloprotease; TGF: Transforming growth factor; VEGF: Vascular endothelial growth factor.

## Competing interests

The authors declare that they have no competing interests.

## Authors’ contributions

TK, PD and EP conceived and designed the experiments. The experiments were performed by TK, SK, NT and HL. Paper was written by TK and EP. All authors read and approved the final manuscript.

## Pre-publication history

The pre-publication history for this paper can be accessed here:

http://www.biomedcentral.com/1471-2407/13/518/prepub

## References

[B1] Parkin DM Bray F Ferlay J Pisani P Global cancer statistics, 2002 CA Cancer J Clin 2005 55 74 108 10.3322/canjclin.55.2.74 15761078

[B2] Khong TL Larsen H Raatz Y Paleolog E Angiogenesis as a therapeutic target in arthritis: learning the lessons of the colorectal cancer experience Angiogenesis 2007 10 243 258 10.1007/s10456-007-9081-1 17805984

[B3] Thairu N Kiriakidis S Dawson P Paleolog E Angiogenesis as a therapeutic target in arthritis in 2011: learning the lessons of the colorectal cancer experience Angiogenesis 2011 Epub ahead of print 10.1007/s10456-011-9208-2 21431303

[B4] Mandriota SJ Turner KJ Davies DR Murray PG Morgan NV Sowter HM Wykoff CC Maher ER Harris AL Ratcliffe PJ Maxwell PH HIF activation identifies early lesions in VHL kidneys: evidence for site-specific tumor suppressor function in the nephron Cancer Cell 2002 1 459 468 10.1016/S1535-6108(02)00071-5 12124175

[B5] Goethals L Debucquoy A Perneel C Geboes K Ectors N De Schutter H Penninckx F McBride WH Begg AC Haustermans KM Hypoxia in human colorectal adenocarcinoma: comparison between extrinsic and potential intrinsic hypoxia markers Int J Radiat Oncol Biol Phys 2006 65 246 254 10.1016/j.ijrobp.2006.01.007 16618579

[B6] Semenza G Signal transduction to hypoxia-inducible factor 1 Biochem Pharmacol 2002 64 993 998 10.1016/S0006-2952(02)01168-1 12213597

[B7] Lee JW Bae SH Jeong JW Kim SH Kim KW Hypoxia-inducible factor (HIF-1)alpha: its protein stability and biological functions Exp Mol Med 2004 36 1 12 10.1038/emm.2004.1 15031665

[B8] Epstein AC Gleadle JM McNeill LA Hewitson KS O'Rourke J Mole DR Mukherji M Metzen E Wilson MI Dhanda A C. elegans EGL-9 and mammalian homologs define a family of dioxygenases that regulate HIF by prolyl hydroxylation Cell 2001 107 43 54 10.1016/S0092-8674(01)00507-4 11595184

[B9] Ivan M Haberberger T Gervasi DC Michelson KS Gunzler V Kondo K Yang H Sorokina I Conaway RC Conaway JW Kaelin WG Jr Biochemical purification and pharmacological inhibition of a mammalian prolyl hydroxylase acting on hypoxia-inducible factor Proc Natl Acad Sci USA 2002 99 13459 13464 10.1073/pnas.192342099 12351678 PMC129695

[B10] Bracken CP Fedele AO Linke S Balrak W Lisy K Whitelaw ML Peet DJ Cell-specific regulation of hypoxia-inducible factor (HIF)-1alpha and HIF-2alpha stabilization and transactivation in a graded oxygen environment J Biol Chem 2006 281 22575 22585 10.1074/jbc.M600288200 16760477

[B11] Giles RH Lolkema MP Snijckers CM Belderbos M van der Groep P Mans DA van Beest M van Noort M Goldschmeding R van Diest PJ Interplay between VHL/HIF1alpha and Wnt/beta-catenin pathways during colorectal tumorigenesis Oncogene 2006 25 3065 3070 10.1038/sj.onc.1209330 16407833

[B12] Simiantonaki N Taxeidis M Jayasinghe C Kurzik-Dumke U Kirkpatrick CJ Hypoxia-inducible factor 1 alpha expression increases during colorectal carcinogenesis and tumor progression BMC Cancer 2008 8 320 10.1186/1471-2407-8-320 18983642 PMC2584660

[B13] Kuwai T Kitadai Y Tanaka S Onogawa S Matsutani N Kaio E Ito M Chayama K Expression of hypoxia-inducible factor-1alpha is associated with tumor vascularization in human colorectal carcinoma Int J Cancer 2003 105 176 181 10.1002/ijc.11068 12673675

[B14] Mizukami Y Li J Zhang X Zimmer MA Iliopoulos O Chung DC Hypoxia-inducible factor-1-independent regulation of vascular endothelial growth factor by hypoxia in colon cancer Cancer Res 2004 64 1765 1772 10.1158/0008-5472.CAN-03-3017 14996738

[B15] Rasheed S Harris AL Tekkis PP Turley H Silver A McDonald PJ Talbot IC Glynne-Jones R Northover JM Guenther T Hypoxia-inducible factor-1alpha and -2alpha are expressed in most rectal cancers but only hypoxia-inducible factor-1alpha is associated with prognosis Br J Cancer 2009 100 1666 1673 10.1038/sj.bjc.6605026 19436307 PMC2696753

[B16] Imamura T Kikuchi H Herraiz MT Park DY Mizukami Y Mino-Kenduson M Lynch MP Rueda BR Benita Y Xavier RJ Chung DC HIF-1alpha and HIF-2alpha have divergent roles in colon cancer Int J Cancer 2009 124 763 771 10.1002/ijc.24032 19030186 PMC2682346

[B17] Yoshimura H Dhar DK Kohno H Kubota H Fujii T Ueda S Kinugasa S Tachibana M Nagasue N Prognostic impact of hypoxia-inducible factors 1alpha and 2alpha in colorectal cancer patients: correlation with tumor angiogenesis and cyclooxygenase-2 expression Clin Cancer Res 2004 10 8554 8560 10.1158/1078-0432.CCR-0946-03 15623639

[B18] Cleven AH van Engeland M Wouters BG de Bruine AP Stromal expression of hypoxia regulated proteins is an adverse prognostic factor in colorectal carcinomas Cell Oncol 2007 29 229 240 17452775 10.1155/2007/945802 PMC4617795

[B19] Lockhart AC Berlin JD The epidermal growth factor receptor as a target for colorectal cancer therapy Semin Oncol 2005 32 52 60 10.1053/j.seminoncol.2004.09.036 15726506

[B20] Akagi M Kawaguchi M Liu W McCarty MF Takeda A Fan F Stoeltzing O Parikh AA Jung YD Bucana CD Induction of neuropilin-1 and vascular endothelial growth factor by epidermal growth factor in human gastric cancer cells Br J Cancer 2003 88 796 802 10.1038/sj.bjc.6600811 12618892 PMC2376351

[B21] Okamura K Morimoto A Hamanaka R Ono M Kohno K Uchida Y Kuwano M A model system for tumor angiogenesis: involvement of transforming growth factor-alpha in tube formation of human microvascular endothelial cells induced by esophageal cancer cells Biochem Biophys Res Commun 1992 186 1471 1479 10.1016/S0006-291X(05)81572-4 1380804

[B22] Goldman CK Kim J Wong WL King V Brock T Gillespie GY Epidermal growth factor stimulates vascular endothelial growth factor production by human malignant glioma cells: a model of glioblastoma multiforme pathophysiology Mol Biol Cell 1993 4 121 133 10.1091/mbc.4.1.121 7680247 PMC300905

[B23] Perrotte P Matsumoto T Inoue K Kuniyasu H Eve BY Hicklin DJ Radinsky R Dinney CP Anti-epidermal growth factor receptor antibody C225 inhibits angiogenesis in human transitional cell carcinoma growing orthotopically in nude mice Clin Cancer Res 1999 5 257 265 10037173

[B24] Petit AM Rak J Hung MC Rockwell P Goldstein N Fendly B Kerbel RS Neutralizing antibodies against epidermal growth factor and ErbB-2/neu receptor tyrosine kinases down-regulate vascular endothelial growth factor production by tumor cells in vitro and in vivo: angiogenic implications for signal transduction therapy of solid tumors Am J Pathol 1997 151 1523 1530 9403702 PMC1858348

[B25] Ciardiello F Caputo R Bianco R Damiano V Fontanini G Cuccato S De Placido S Bianco AR Tortora G Inhibition of growth factor production and angiogenesis in human cancer cells by ZD1839 (Iressa), a selective epidermal growth factor receptor tyrosine kinase inhibitor Clin Cancer Res 2001 7 1459 1465 11350918

[B26] Francoual M Etienne-Grimaldi MC Formento JL Benchimol D Bourgeon A Chazal M Letoublon C Andre T Gilly N Delpero JR EGFR in colorectal cancer: more than a simple receptor Ann Oncol 2006 17 962 967 10.1093/annonc/mdl037 16524971

[B27] Johnston JB Navaratnam S Pitz MW Maniate JM Wiechec E Baust H Gingerich J Skliris GP Murphy LC Los M Targeting the EGFR pathway for cancer therapy Curr Med Chem 2006 13 3483 3492 10.2174/092986706779026174 17168718

[B28] Van Cutsem E Peeters M Siena S Humblet Y Hendlisz A Neyns B Canon JL Van Laethem JL Maurel J Richardson G Open-label phase III trial of panitumumab plus best supportive care compared with best supportive care alone in patients with chemotherapy-refractory metastatic colorectal cancer J Clin Oncol 2007 25 1658 1664 10.1200/JCO.2006.08.1620 17470858

[B29] Richard DE Berra E Gothie E Roux D Pouyssegur J p42/p44 mitogen-activated protein kinases phosphorylate hypoxia-inducible factor 1alpha (HIF-1alpha) and enhance the transcriptional activity of HIF-1 J Biol Chem 1999 274 32631 32637 10.1074/jbc.274.46.32631 10551817

[B30] Shafee N Kaluz S Ru N Stanbridge EJ PI3K/Akt activity has variable cell-specific effects on expression of HIF target genes, CA9 and VEGF, in human cancer cell lines Cancer Lett 2009 282 109 115 10.1016/j.canlet.2009.03.004 19342157 PMC2706285

[B31] Laughner E Taghavi P Chiles K Mahon PC Semenza GL HER2 (neu) signaling increases the rate of hypoxia-inducible factor 1alpha (HIF-1alpha) synthesis: novel mechanism for HIF-1-mediated vascular endothelial growth factor expression Mol Cell Biol 2001 21 3995 4004 10.1128/MCB.21.12.3995-4004.2001 11359907 PMC87062

[B32] Peng XH Karna P Cao Z Jiang BH Zhou M Yang L Cross-talk between epidermal growth factor receptor and hypoxia-inducible factor-1alpha signal pathways increases resistance to apoptosis by up-regulating survivin gene expression J Biol Chem 2006 281 25903 25914 10.1074/jbc.M603414200 16847054 PMC3132567

[B33] Zhong H Chiles K Feldser D Laughner E Hanrahan C Georgescu MM Simons JW Semenza GL Modulation of hypoxia-inducible factor 1alpha expression by the epidermal growth factor/phosphatidylinositol 3-kinase/PTEN/AKT/FRAP pathway in human prostate cancer cells: implications for tumor angiogenesis and therapeutics Cancer Res 2000 60 1541 1545 10749120

[B34] Denekamp J Vascular attack as a therapeutic strategy for cancer Cancer Metastasis Rev 1990 9 267 282 10.1007/BF00046365 2292139

[B35] Keese M Magdeburg RJ Herzog T Hasenberg T Offterdinger M Pepperkok R Sturm JW Bastiaens PI Imaging epidermal growth factor receptor phosphorylation in human colorectal cancer cells and human tissues J Biol Chem 2005 280 27826 27831 10.1074/jbc.M504485200 15908435

[B36] Fogh J Fogh JM Orfeo T One hundred and twenty-seven cultured human tumor cell lines producing tumors in nude mice J Natl Cancer Inst 1977 59 221 226 327080 10.1093/jnci/59.1.221

[B37] Livak KJ Schmittgen TD Analysis of relative gene expression data using real-time quantitative PCR and the 2(−Delta Delta C(T)) Method Methods 2001 25 402 408 10.1006/meth.2001.1262 11846609

[B38] Muz B Larsen H Madden L Kiriakidis S Paleolog EM Prolyl hydroxylase domain enzyme 2 is the major player in regulating hypoxic responses in rheumatoid arthritis Arthritis Rheum 2012 64 2856 2867 10.1002/art.34479 22488178

[B39] Larsen H Muz B Khong TL Feldmann M Paleolog EM Differential effects of Th1 versus Th2 cytokines in combination with hypoxia on HIFs and angiogenesis in RA Arthritis Res Ther 2012 14 R180 10.1186/ar3934 22866899 PMC3580575

[B40] Buzzi N Colicheo A Boland R de Boland AR MAP kinases in proliferating human colon cancer Caco-2 cells Mol Cell Biochem 2009 328 201 208 10.1007/s11010-009-0090-9 19301097

[B41] Laprise P Chailler P Houde M Beaulieu JF Boucher MJ Rivard N Phosphatidylinositol 3-kinase controls human intestinal epithelial cell differentiation by promoting adherens junction assembly and p38 MAPK activation J Biol Chem 2002 277 8226 8234 10.1074/jbc.M110235200 11756422

[B42] CRUK (Cancer Research UK) Cancer Stats: Colorectal Cancer 2006

[B43] Folkman J Tumor angiogenesis: therapeutic implications N Engl J Med 1971 285 1182 1186 10.1056/NEJM197111182852108 4938153

[B44] Caro I Boulenc X Rousset M Meunier V Bourrié M Julian B Joyeux H Roques C Berger Y Zweibaum A Fabre G Characterisation of a newly isolated Caco-2 clone (TC-7), as a model of transport processes and biotransformation of drugs Int J Pharm 1995 116 147 158 10.1016/0378-5173(94)00280-I

[B45] Bockmann S Nebe B The in vitro effects of H-89, a specific inhibitor of protein kinase A, in the human colonic carcinoma cell line Caco-2 Eur J Cancer Prev 2003 12 469 478 10.1097/00008469-200312000-00005 14639124

[B46] Wang S Basson MD Identification of functional domains in AKT responsible for distinct roles of AKT isoforms in pressure-stimulated cancer cell adhesion Exp Cell Res 2008 314 286 296 10.1016/j.yexcr.2007.08.005 17825284 PMC2180395

[B47] Liu Y Bodmer WF Analysis of P53 mutations and their expression in 56 colorectal cancer cell lines Proc Natl Acad Sci USA 2006 103 976 981 10.1073/pnas.0510146103 16418264 PMC1327731

[B48] Brink M de Goeij AF Weijenberg MP Roemen GM Lentjes MH Pachen MM Smits KM de Bruine AP Goldbohm RA van den Brandt PA K-ras oncogene mutations in sporadic colorectal cancer in The Netherlands Cohort Study Carcinogenesis 2003 24 703 710 10.1093/carcin/bgg009 12727799

[B49] Kikuchi H Pino MS Zeng M Shirasawa S Chung DC Oncogenic KRAS and BRAF differentially regulate hypoxia-inducible factor-1alpha and -2alpha in colon cancer Cancer Res 2009 69 8499 8506 10.1158/0008-5472.CAN-09-2213 19843849 PMC2811371

[B50] Baba Y Huttenhower C Nosho K Tanaka N Shima K Hazra A Schernhammer ES Hunter DJ Giovannucci EL Fuchs CS Ogino S Epigenomic diversity of colorectal cancer indicated by LINE-1 methylation in a database of 869 tumors Mol Cancer 2010 9 125 10.1186/1476-4598-9-125 20507599 PMC2892454

[B51] Damstrup L Kuwada SK Dempsey PJ Brown CL Hawkey CJ Poulsen HS Wiley HS Coffey RJ Jr Amphiregulin acts as an autocrine growth factor in two human polarizing colon cancer lines that exhibit domain selective EGF receptor mitogenesis Br J Cancer 1999 80 1012 1019 10.1038/sj.bjc.6690456 10362109 PMC2363033

[B52] Yonezawa M Wada K Tatsuguchi A Akamatsu T Gudis K Seo T Mitsui K Nagata K Tanaka S Fujimori S Sakamoto C Heregulin-induced VEGF expression via the ErbB3 signaling pathway in colon cancer Digestion 2009 80 215 225 10.1159/000229775 19797898

[B53] Gentile LB Piva B Diaz BL Hypertonic stress induces VEGF production in human colon cancer cell line Caco-2: inhibitory role of autocrine PGE(2) PLoS One 2011 6 e25193 10.1371/journal.pone.0025193 21980393 PMC3182186

[B54] Bu XD Li N Tian XQ Huang PL Caco-2 and LS174T cell lines provide different models for studying mucin expression in colon cancer Tissue Cell 2011 43 201 206 10.1016/j.tice.2011.03.002 21470648

[B55] Dhawan P Ahmad R Chaturvedi R Smith JJ Midha R Mittal MK Krishnan M Chen X Eschrich S Yeatman TJ Claudin-2 expression increases tumorigenicity of colon cancer cells: role of epidermal growth factor receptor activation Oncogene 2011 30 3234 3247 10.1038/onc.2011.43 21383692 PMC3591522

[B56] de Bruine AP de Vries JE Dinjens WN Moerkerk PT van der Linden EP Pijls MM ten Kate J Bosman FT Human Caco-2 cells transfected with c-Ha-Ras as a model for endocrine differentiation in the large intestine Differentiation 1993 53 51 60 10.1111/j.1432-0436.1993.tb00645.x 8508948

[B57] Zgouras D Wachtershauser A Frings D Stein J Butyrate impairs intestinal tumor cell-induced angiogenesis by inhibiting HIF-1alpha nuclear translocation Biochem Biophys Res Commun 2003 300 832 838 10.1016/S0006-291X(02)02916-9 12559948

[B58] Matsuo Y Sawai H Ma J Xu D Ochi N Yasuda A Takahashi H Funahashi H Takeyama H IL-1alpha secreted by colon cancer cells enhances angiogenesis: the relationship between IL-1alpha release and tumor cells’ potential for liver metastasis J Surg Oncol 2009 99 361 367 10.1002/jso.21245 19204921

[B59] Franovic A Holterman CE Payette J Lee S Human cancers converge at the HIF-2alpha oncogenic axis Proc Natl Acad Sci USA 2009 106 21306 21311 10.1073/pnas.0906432106 19955413 PMC2795516

[B60] Richard DE Berra E Pouyssegur J Nonhypoxic pathway mediates the induction of hypoxia-inducible factor 1alpha in vascular smooth muscle cells J Biol Chem 2000 275 26765 26771 10837481 10.1074/jbc.M003325200

[B61] Pore N Jiang Z Gupta A Cerniglia G Kao GD Maity A EGFR tyrosine kinase inhibitors decrease VEGF expression by both hypoxia-inducible factor (HIF)-1-independent and HIF-1-dependent mechanisms Cancer Res 2006 66 3197 3204 10.1158/0008-5472.CAN-05-3090 16540671

[B62] Sang N Stiehl DP Bohensky J Leshchinsky I Srinivas V Caro J MAPK signaling up-regulates the activity of hypoxia-inducible factors by its effects on p300 J Biol Chem 2003 278 14013 14019 10.1074/jbc.M209702200 12588875 PMC4518846

[B63] Mahon PC Hirota K Semenza GL FIH-1: a novel protein that interacts with HIF-1alpha and VHL to mediate repression of HIF-1 transcriptional activity Genes Dev 2001 15 2675 2686 10.1101/gad.924501 11641274 PMC312814

[B64] Katoh Y Katoh M Comparative integromics on Angiopoietin family members Int J Mol Med 2006 17 1145 1149 16685428

[B65] Galaup A Cazes A Le Jan S Philippe J Connault E Le Coz E Mekid H Mir LM Opolon P Corvol P Angiopoietin-like 4 prevents metastasis through inhibition of vascular permeability and tumor cell motility and invasiveness Proc Natl Acad Sci USA 2006 103 18721 18726 10.1073/pnas.0609025103 17130448 PMC1693729

[B66] Le Jan S Amy C Cazes A Monnot C Lamande N Favier J Philippe J Sibony M Gasc JM Corvol P Germain S Angiopoietin-like 4 is a proangiogenic factor produced during ischemia and in conventional renal cell carcinoma Am J Pathol 2003 162 1521 1528 10.1016/S0002-9440(10)64285-X 12707035 PMC1851201

[B67] Oike Y Ito Y Maekawa H Morisada T Kubota Y Akao M Urano T Yasunaga K Suda T Angiopoietin-related growth factor (AGF) promotes angiogenesis Blood 2004 103 3760 3765 10.1182/blood-2003-04-1272 14764539

[B68] Nakayama T Hirakawa H Shibata K Nazneen A Abe K Nagayasu T Taguchi T Expression of angiopoietin-like 4 (ANGPTL4) in human colorectal cancer: ANGPTL4 promotes venous invasion and distant metastasis Oncol Rep 2011 25 929 935 21308352 10.3892/or.2011.1176

[B69] Merlos-Suarez A Batlle E Eph-ephrin signalling in adult tissues and cancer Curr Opin Cell Biol 2008 20 194 200 10.1016/j.ceb.2008.01.011 18353626

[B70] Surawska H Ma PC Salgia R The role of ephrins and Eph receptors in cancer Cytokine Growth Factor Rev 2004 15 419 433 10.1016/j.cytogfr.2004.09.002 15561600

[B71] Brantley DM Cheng N Thompson EJ Lin Q Brekken RA Thorpe PE Muraoka RS Cerretti DP Pozzi A Jackson D Soluble Eph A receptors inhibit tumor angiogenesis and progression in vivo Oncogene 2002 21 7011 7026 10.1038/sj.onc.1205679 12370823

[B72] Hafner C Schmitz G Meyer S Bataille F Hau P Langmann T Dietmaier W Landthaler M Vogt T Differential gene expression of Eph receptors and ephrins in benign human tissues and cancers Clin Chem 2004 50 490 499 10.1373/clinchem.2003.026849 14726470

[B73] Batlle E Bacani J Begthel H Jonkheer S Gregorieff A van de Born M Malats N Sancho E Boon E Pawson T EphB receptor activity suppresses colorectal cancer progression Nature 2005 435 1126 1130 10.1038/nature03626 15973414

[B74] Javelaud D Mauviel A Mammalian transforming growth factor-betas: Smad signaling and physio-pathological roles Int J Biochem Cell Biol 2004 36 1161 1165 10.1016/S1357-2725(03)00255-3 15109563

[B75] Robson H Anderson E James RD Schofield PF Transforming growth factor beta 1 expression in human colorectal tumours: an independent prognostic marker in a subgroup of poor prognosis patients Br J Cancer 1996 74 753 758 10.1038/bjc.1996.432 8795578 PMC2074698

[B76] Gulubova M Manolova I Ananiev J Julianov A Yovchev Y Peeva K Role of TGF-beta1, its receptor TGFbetaRII, and Smad proteins in the progression of colorectal cancer Int J Colorectal Dis 2010 25 591 599 10.1007/s00384-010-0906-9 20165854

[B77] Berger AP Kofler K Bektic J Rogatsch H Steiner H Bartsch G Klocker H Increased growth factor production in a human prostatic stromal cell culture model caused by hypoxia Prostate 2003 57 57 65 10.1002/pros.10279 12886524

[B78] Xiong B Gong LL Zhang F Hu MB Yuan HY TGF beta1 expression and angiogenesis in colorectal cancer tissue World J Gastroenterol 2002 8 496 498 12046078 10.3748/wjg.v8.i3.496 PMC4656429

[B79] Ono M Kuwano M Molecular mechanisms of epidermal growth factor receptor (EGFR) activation and response to gefitinib and other EGFR-targeting drugs Clin Cancer Res 2006 12 7242 7251 10.1158/1078-0432.CCR-06-0646 17189395

[B80] Fujimoto K Sheng H Shao J Beauchamp RD Transforming growth factor-beta1 promotes invasiveness after cellular transformation with activated Ras in intestinal epithelial cells Exp Cell Res 2001 266 239 249 10.1006/excr.2000.5229 11399052

[B81] Bowman T Garcia R Turkson J Jove R STATs in oncogenesis Oncogene 2000 19 2474 2488 10.1038/sj.onc.1203527 10851046

[B82] Testa JR Bellacosa A AKT plays a central role in tumorigenesis Proc Natl Acad Sci USA 2001 98 10983 10985 10.1073/pnas.211430998 11572954 PMC58668

[B83] Zhou BP Liao Y Xia W Spohn B Lee MH Hung MC Cytoplasmic localization of p21Cip1/WAF1 by Akt-induced phosphorylation in HER-2/neu-overexpressing cells Nat Cell Biol 2001 3 245 252 10.1038/35060032 11231573

[B84] Chan TO Rittenhouse SE Tsichlis PN AKT/PKB and other D3 phosphoinositide-regulated kinases: kinase activation by phosphoinositide-dependent phosphorylation Annu Rev Biochem 1999 68 965 1014 10.1146/annurev.biochem.68.1.965 10872470

[B85] Thant AA Nawa A Kikkawa F Ichigotani Y Zhang Y Sein TT Amin AR Hamaguchi M Fibronectin activates matrix metalloproteinase-9 secretion via the MEK1-MAPK and the PI3K-Akt pathways in ovarian cancer cells Clin Exp Metastasis 2000 18 423 428 10.1023/A:1010921730952 11467775

[B86] Engebraaten O Bjerkvig R Pedersen PH Laerum OD Effects of EGF, bFGF, NGF and PDGF(bb) on cell proliferative, migratory and invasive capacities of human brain-tumour biopsies in vitro Int J Cancer 1993 53 209 214 10.1002/ijc.2910530206 8381111

[B87] Bokemeyer C Van Cutsem E Rougier P Ciardiello F Heeger S Schlichting M Celik I Kohne CH Addition of cetuximab to chemotherapy as first-line treatment for KRAS wild-type metastatic colorectal cancer: pooled analysis of the CRYSTAL and OPUS randomised clinical trials Eur J Cancer 2012 48 1466 1475 10.1016/j.ejca.2012.02.057 22446022

[B88] Van Cutsem E Kohne CH Hitre E Zaluski J Chang Chien CR Makhson A D'Haens G Pinter T Lim R Bodoky G Cetuximab and chemotherapy as initial treatment for metastatic colorectal cancer N Engl J Med 2009 360 1408 1417 10.1056/NEJMoa0805019 19339720

[B89] Thairu N Kiriakidis S Dawson P Paleolog E HIF-Isoforms have divergent roles in the angiogenesis of colorectal cancer Colorectal Dis 2011 13 20 (P034) 10.1007/s10456-011-9208-2 21431303

[B90] Thairu N Kiriakidis S Dawson P Paleolog E Short-term cultures of tumour-derived colorectal cancer cells – a novel in vitro model for the evaluation of angiogenesis in colorectal cancer Br J Surg 2012 99 7 (abstract O637)

[B91] Thairu N Kiriakidis S Dawson P Paleolog E Short-term cultures of tumour-derived colorectal cancer cells – a novel in vitro model for the evaluation of angiogenesis in colorectal cancer Colorectal Dis 2012 14 16 (P027)

